# Multimodality Imaging of Intramyocardial Dissecting Hematoma: A Rare, Potentially Fatal Complication of Percutaneous Coronary Intervention

**DOI:** 10.1016/j.jaccas.2025.106598

**Published:** 2026-01-21

**Authors:** Yvonne Johanna Maria van Cauteren, Martijn Willem Smulders, Casper Mihl, Suzanne Gommers, Geertruida Petronella Bijvoet, Árpád Lux, Ralph Antonius Leonardus Josephus Theunissen

**Affiliations:** a Department of Cardiology, Maastricht UMC+, Maastricht, the Netherlands; b Department of Radiology and Nuclear Medicine, Maastricht UMC+, Maastricht, the Netherlands; c Cardiovascular Research Institute Maastricht (CARIM), Maastricht University, Maastricht, the Netherlands

**Keywords:** cardiac magnetic resonance, computed tomography angiography, echocardiography, percutaneous coronary intervention

## Abstract

**Background:**

Intramyocardial dissecting hematoma is a rare complication of percutaneous coronary intervention.

**Case Summary:**

A 56-year-old patient underwent percutaneous coronary intervention with successful right coronary artery stenting, complicated by distal wire perforation and contrast extravasation. Although initially managed conservatively, follow-up echocardiography showed a rapidly expanding septal intramyocardial hematoma. Urgent coronary angiography demonstrated multiple sites of contrast accumulation, which progressed despite balloon inflation, but ultimately stabilized after covered stent placement. Cardiovascular magnetic resonance and computed tomography angiography confirmed the diagnosis of intramyocardial hematoma and revealed transmural myocardial infarction with microvascular obstruction.

**Discussion:**

Most reports describe intramyocardial hematomas in chronic total occlusion or prior bypass surgery. In this case, multimodality imaging suggests subclinical myocardial infarction with spontaneous reperfusion, creating a vulnerable environment for rapid hematoma expansion. Covered stent placement likely prevented septal wall rupture.

**Take-Home Message:**

Multimodality imaging facilitates diagnosing intramural hematoma, a rare but potentially life-threatening complication.

## Visual Summary

**Figure:**
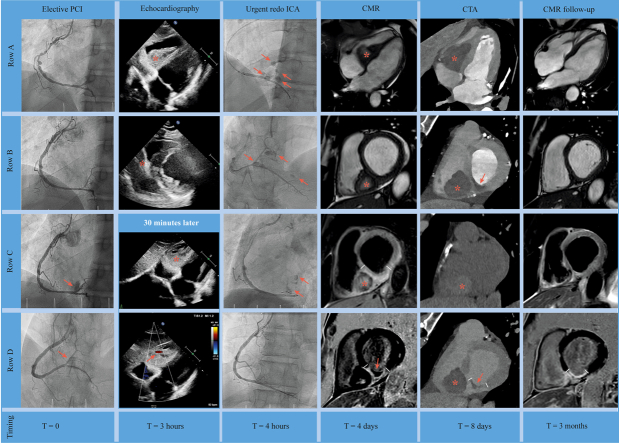

**Visual Summary:**
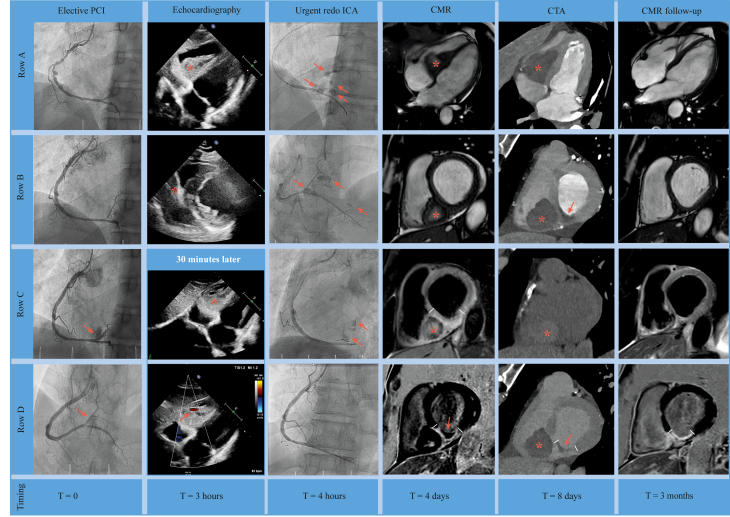
Multimodality Imaging of Intramyocardial Dissecting Hematoma The first column shows elective PCI (T = 0): row A = coronary angiography of RCA before stenting, row B = result after placement of 2 drug-eluting stents, and rows C and D = contrast extravasation at 1 site (arrow). The second column shows echocardiography (T = 3 hours): row A = subcostal view showing septal intramyocardial mass with partial occlusion of the right ventricle (asterisk), row B = parasternal short-axis view showing septal intramyocardial mass (asterisk), and rows C and D = repeated echocardiography after 30 minutes, showing subcostal view with rapid progression of intramyocardial mass (asterisk), with suggestion of flow inside this mass based on color Doppler imaging (arrow). The third column shows urgent redo ICA (T = 4 hours): rows A and B = urgent coronary angiography (2nd procedure) showing contrast extravasation at multiple sites (arrow), row C = persistent contrast extravasation despite prolonged balloon inflations (arrow), and row D = covered stent placement sealed the perforation and ended the extravasation. The fourth column shows CMR (T = 4 days): rows A and B = long-axis 4-chamber and short-axis cine images showing intramyocardial hematoma (asterisk) with partial (not obstructive) occlusion of the right ventricle, row C = short-axis T2-weighted imaging showing hyperintensity consistent with edema (between lines) and intramyocardial hematoma (asterisk); and row D = short-axis late enhancement imaging showing transmural hyperenhancement consistent with myocardial infarction inferior (between lines) and microvascular obstruction (MVO) (arrow). The fifth column shows CTA (T = 8 days): row A = long-axis 4-chamber view after intravenous contrast injection, showing large intramyocardial hematoma (asterisk) without active contrast leakage; row B = corresponding short-axis view showing large intramyocardial hematoma (asterisk) and signs of MVO (arrow), row C = native scan (without iodin contrast agent) short-axis view showing increased signal consistent with hematoma (asterisk), and row D = short-axis late enhancement imaging showing large intramyocardial hematoma (asterisk) and signs of transmural myocardial infarction (between lines) and MVO (arrow). The last column shows CMR (T = 3 months): comparable images with fourth column, showing near-complete resolution of intramyocardial hematoma (rows A and B), no more signs of edema (row C), and chronic transmural infarction (between lines) without MVO (row D). CMR = cardiac magnetic resonance; CTA = computed tomography angiography; ICA = invasive coronary angiography; PCI = percutaneous coronary intervention.

## History of Presentation

A 56-year-old patient without history of cardiovascular disease presented to the outpatient clinic with a sudden onset of exercise-induced chest pain. Echocardiography showed global left ventricular dysfunction and severe hypokinesia of the inferior and inferolateral segments. Coronary angiography revealed a 90% stenosis and a focal saccular aneurysm of the mid segment of the right coronary artery (RCA). The left coronary arteries showed borderline stenoses that were not hemodynamically significant (fractional flow reserve negative). After discussion in the multidisciplinary heart team, an elective optical coherence tomography (OCT)–guided percutaneous coronary intervention (PCI) of the RCA was conducted. For OCT imaging, a Dragonfly catheter (Abbott) with a guide extension (Telescope) was used. OCT revealed thin- and thick-cap fibroatheromas, macrophage infiltration, and fibrocalcific plaques with >180^◦^ calcification. Subsequently, PCI was performed using a Judkins Right 4.0 catheter (Cordis) and SION (Asahi) guidewire. Two drug-eluting stents (Synergy XD 3.5 × 38 mm/3.0 × 32 mm [Boston Scientific]) were placed in the proximal and distal RCA segments. Post-PCI OCT confirmed good stent apposition and satisfactory expansion. At the end of the procedure, contrast extravasation and a small intramural hematoma were observed, consistent with an Ellis type 2 perforation (Video 1). Echocardiography was repeatedly performed during and at the end of the procedure, showing no pericardial effusion. The patient was transferred to the cardiac care unit with minimal chest pain, and remained hemodynamically stable during observation. However, approximately 3 hours later, a sudden progression of chest pain was noted.

**Take-Home Messages**

• Given the potential for delayed pericardial effusion or intramyocardial hematoma in the setting of contrast extravasation, close serial echocardiography monitoring should be considered after coronary perforation, even when no effusion is initially apparent.
• Multimodality imaging is valuable for the diagnosis, management, and follow-up of patients with complications after PCI.
• In the case of a large intramyocardial dissecting hematoma, covered stent placement may prevent septal rupture by halting active extravasation.

## Differential Diagnosis

In the evaluation of chest pain after PCI, several differential diagnoses should be considered. Most commonly, the pain is benign, resulting from local vessel stretch and adventitial injury. However, more serious etiologies include distal embolization of plaque or thrombus (typically identified intraprocedurally), coronary vasospasm, stent edge dissection, early in-stent thrombosis, and pericardial effusion.^1^ In this case, the primary concern was pericardial effusion, given the previously observed contrast extravasation.

## Investigations

In the cardiac care unit, follow-up transthoracic echocardiography was performed immediately after the sudden progression of chest pain. No pericardial effusion was observed; however, a large intracardiac mass was identified within the right ventricle, attached to the basal/mid interventricular septum (Videos 2 and 3). The differential diagnosis included intramyocardial hematoma or right ventricular thrombus without inflow or outflow obstruction. Repeated echocardiography after 30 minutes demonstrated an increase in mass size, with cavitations and apparent flow on color Doppler imaging, highly suggestive of intramyocardial hematoma (Video 4, Video 5, Video 6). The progression raised concern for active bleeding from the coronary arteries into the hematoma.

## Management

Urgent coronary angiography was performed using the same type of guiding catheter and wire as the initial procedure. Multiple sites of contrast accumulation within the interventricular septum were seen, confirming the working diagnosis (Videos 7 and 8). Prolonged balloon inflation at various sites along the distal RCA and right posterolateral (RPL) region did not seal the perforation (Video 9). Contralateral injections of the left coronary arteries did not show any vascular communication with right-sided contrast extravasation. During the procedure, the patient became hemodynamically unstable. After multidisciplinary consultation between cardiothoracic surgeons, interventional cardiologists, and imaging cardiologists, a covered stent (Biotronik 3.0 × 20 mm) was placed in the distal RCA covering the RPL bifurcation, successfully sealing the perforation and terminating the contrast extravasation (Video 10). A small RPL branch was occluded, resulting in transient chest pain and ST-segment elevations on electrocardiogram, which was managed conservatively. After stent placement, the patient's hemodynamic status stabilized, averting the need for surgical intervention or extracorporeal membrane oxygenation.

## Outcome and Follow-Up

Serial echocardiography examinations over the following hours and days showed no progression. Cardiac magnetic resonance (CMR) was performed 4 days later, showing an intramyocardial hematoma within the interventricular septum, extending toward the right ventricle. A thin myocardial layer overlying the hematoma was visible, supporting the diagnosis of intramyocardial hematoma rather than thrombus. Tissue characteristics across various sequences were consistent with the subacute phase. Assessment of rest perfusion to evaluate residual leakage proved challenging. Due to hemoglobin degradation products (eg methemoglobin) within the hematoma, high signal intensity was already present on T_1_-weighted images before any Gadovist (gadobutrol) arrived in the hematoma. However, an acute inferior myocardial infarction could be identified, with hyperintensity on T_2_-weighted imaging, transmural late gadolinium enhancement with extension to the basal right ventricular inferior wall, and microvascular obstruction (MVO). Computed tomography angiography (CTA) was performed 8 days post-PCI, with no evidence of ongoing contrast extravasation. CTA showed comparable findings consistent with a couple days’ old intramyocardial hematoma, and signs of acute myocardial infarction with MVO on late enhancement imaging. During hospitalization, the patient developed transient third-degree atrioventricular block, which resolved spontaneously without intervention. The patient was discharged 13 days after the initial PCI. During outpatient visits, the patient reported no recurrent chest pain or symptoms of heart failure. Follow-up CMR performed 3 months after the PCI demonstrated almost complete resolution of the intramyocardial dissecting hematoma.

## Discussion

This case report describes a patient who developed a giant septal intramyocardial dissecting hematoma after PCI. Intramyocardial hematoma is a rare but potentially fatal complication of PCI and (sub)acute myocardial infarction.^2^ A recognized mechanism involves bleeding-induced dissection between myocardial layers. Progressive hematoma expansion may eventually lead to myocardial wall rupture. Other complications include (pseudo)tamponade and compression of epicardial coronary arteries, particularly when the hematoma is located subepicardial.^3^ Most case reports involve patients with chronic total occlusion,^4^ likely due to the used techniques and more aggressive guidewires. It has also been described in patients with prior coronary artery bypass grafting,^3^ where obliteration of the pericardial space by fibrotic adhesions may redirect bleeding between epicardial and myocardial layers, resulting in an intramyocardial dissecting hematoma.^5^

Although wire perforation likely contributed to the development of intramyocardial hematoma in this patient, noninvasive imaging suggests a preceding subclinical myocardial infarction with spontaneous reperfusion. Other findings supporting this differential diagnosis include recent-onset chest pain, hypokinesia of inferior segments on echocardiography, and OCT findings consistent with vulnerable plaque morphology. Moreover, CMR demonstrated transmural myocardial infarction with edema and MVO—features typically occurring in recent (<1 month) myocardial infarctions.^6^^,^^7^ The location of the infarct visualized on CMR and CTA could not be attributed to occlusion of the RPL branch resulting from the placement of a covered stent.

We hypothesize that the recent myocardial infarction predisposed the myocardium to increased vulnerability, and that the RCA perforation with contrast extravasation in this context resulted in uncontrolled hematoma expansion and impending septal wall rupture. Urgent PCI with covered stent placement likely prevented further clinical deterioration.

Ellis type I (extraluminal crater without extravasation) or type II (pericardial or myocardial blush without contrast jet extravasation) coronary artery perforations are often managed conservatively. Prolonged balloon inflation may seal the perforation. If unsuccessful, covered stent placement is indicated. Alternative treatments include local delivery of subcutaneous fat, thrombin injection, or occlusive coils or beads.^8^^,^^9^ Protamine may be used in unstable patients to reverse heparin effects, but carries a slight risk of coronary and peripheral arterial thrombosis.^10^ In this case, protamine was withheld because the patient had undergone coronary stent placement and showed no pericardial effusion during 30 minutes of observation. In retrospect, earlier echocardiography during observation in the cardiac care unit may have been warranted despite the hemodynamic stable situation. This case underscores that even Ellis type I and II perforations can lead to significant morbidity and mortality. Prompt recognition, close monitoring with repeated echocardiography, and timely intervention are essential to minimize further harm.

## Conclusions

This case report highlights intramyocardial dissecting hematoma as a potentially life-threatening complication requiring prompt recognition. Using a multimodal imaging approach, combining noninvasive and invasive techniques, enabled effective symptom management and likely prevented septal wall rupture through covered stent placement.

## Funding Support and Author Disclosures

This work was supported by an academic grant (Maastricht UMC+ 2023 to Dr Smulders en dr Mihl). Dr Smulders reports speaking fees for Daiichi Sankyo Europe (ie, ESC highlights “21/”22). Dr Mihl reports speaking fees for Bayer Healthcare. Dr Smulders en dr Mihl have received funding (2024) for a collaborative project cofinanced with PPP allowance made available by Health-Holland, Top Sector Life Sciences & Health to stimulate public-private partnerships and funding from Siemens Healthineers Nederland B.V. All of these are not related to this study. All other authors have reported that they have no relationships relevant to the contents of this paper to disclose.

## Appendix

Video 1 Percutaneous Coronary Intervention Left anterior oblique cranial view of the right coronary artery at the end of elective procedure, showing contrast extravasation of the right posterolateral branch.

Video 2 Echocardiography Subcostal view showing a septal intramyocardial mass with partial occlusion of the right ventricle.

Video 3 Echocardiography Four-chamber view showing a septal intramyocardial mass.

Video 4 Repeated Echocardiography Subcostal view showing increase of the septal intramyocardial mass with cavitations.

Video 5 Repeated Echocardiography Subcostal view with color Doppler imaging, with suggestion of flow inside the septal intramyocardial mass.

Video 6 Repeated Echocardiography Four-chamber view showing increase of septal intramyocardial mass with a small cavitation.

Video 7 Urgent Coronary Angiography Right anterior oblique cranial view of the right coronary artery, showing contrast extravasation at the distal right coronary artery and posterolateral branch region.

Video 8 Urgent Coronary Angiography Left anterior oblique cranial view of the right coronary artery, showing an increase of contrast extravasation compared with the first procedure, with contrast extravasation at multiple sites.

Video 9 Urgent Coronary Angiography: Balloon Inflation Left anterior oblique cranial view of the right coronary artery, showing an increase in contrast extravasation sites despite prolonged balloon inflation.

Video 10 Urgent Coronary Angiography: Covered Stent Placing Left anterior oblique cranial view of the right coronary artery, showing no more contrast extravasation after placement of covered stent in the distal right coronary artery, covering the right posterolateral branch.

## References

[1] Kini A.S., Lee P., Mitre C.A., Duffy M.E., Sharma S.K. (2003). Postprocedure chest pain after coronary stenting: implications on clinical restenosis. J Am Coll Cardiol.

[2] Rossi P.M., de Abreu M., Reyes G. (2022). Intramyocardial dissecting hematoma: a mechanical complication needing surgical therapy?. JACC Case Rep.

[3] Ezad S., Wardill T., Talwar S. (2022). Intramyocardial haematoma complicating chronic total occlusion percutaneous coronary intervention: case series and review of the literature. Cardiovasc Revasc Med.

[4] van Hattem V.A.E., Otterspoor L.C., Lipsic E., Vlaar P.J.J. (2022). Right ventricular hematoma: a rare but potentially fatal complication of percutaneous coronary artery intervention. Catheter Cardiovasc Interv.

[5] Slootweg A.P., Louwerenburg J.W., Mecozzi G., Wagenaar L.J., Verhorst P.M. (2012). Obstructive intramyocardial haematoma after percutaneous coronary intervention. Neth Heart J.

[6] Smulders M.W., Bekkers S.C., Kim H.W. (2015). Performance of CMR methods for differentiating acute from chronic MI. JACC Cardiovasc Imaging.

[7] Wu K.C. (2012). CMR of microvascular obstruction and hemorrhage in myocardial infarction. J Cardiovasc Magn Reson.

[8] Giannini F., Candilio L., Mitomo S. (2018). A practical approach to the management of complications during percutaneous coronary intervention. JACC Cardiovasc Interv.

[9] Kumar S., Al-Ogaili A., Hall A. (2025). Update on the diagnosis and treatment of coronary complications of percutaneous coronary interventions. J Invasive Cardiol.

[10] Alrayes H., Alsaadi A., Alkhatib A. (2025). Safety and complications associated with the use of protamine in percutaneous coronary intervention. J Invasive Cardiol.

